# Effect of T-DNA Integration on Growth of Transgenic *Populus* × *euramericana* cv. Neva Underlying Field Stands

**DOI:** 10.3390/ijms241612952

**Published:** 2023-08-19

**Authors:** Zijie Zhang, Yali Huang, Yan Dong, Yachao Ren, Kejiu Du, Jinmao Wang, Minsheng Yang

**Affiliations:** 1 Institute of Forest Biotechnology, Forestry College, Hebei Agricultural University, Baoding 071000, China; 2 Hebei Key Laboratory for Tree Genetic Resources and Forest Protection, Baoding 071000, China

**Keywords:** genetic transformation, unintended effect, *Populus* × *euramericana* cv. Neva, safety assessment

## Abstract

Multigene cotransformation has been widely used in the study of genetic improvement in crops and trees. However, little is known about the unintended effects and causes of multigene cotransformation in poplars. To gain insight into the unintended effects of T-DNA integration during multigene cotransformation in field stands, here, three lines (A1–A3) of *Populus* × *euramericana* cv. Neva (*PEN*) carrying *Cry1Ac*-*Cry3A*-*BADH* genes and three lines (B1–B3) of *PEN* carrying *Cry1Ac*-*Cry3A*-*NTHK1* genes were used as research objects, with non-transgenic *PEN* as the control. Experimental stands were established at three common gardens in three locations and next generation sequencing (NGS) was used to identify the insertion sites of exogenous genes in six transgenic lines. We compared the growth data of the transgenic and control lines for four consecutive years. The results demonstrated that the tree height and diameter at breast height (DBH) of transgenic lines were significantly lower than those of the control, and the adaptability of transgenic lines in different locations varied significantly. The genotype and the experimental environment showed an interaction effect. A total of seven insertion sites were detected in the six transgenic lines, with B3 having a double-site insertion and the other lines having single copies. There are four insertion sites in the gene region and three insertion sites in the intergenic region. Analysis of the bases near the insertion sites showed that AT content was higher than the average chromosome content in four of the seven insertion sites within 1000 bp. Transcriptome analysis suggested that the differential expression of genes related to plant hormone transduction and lignin synthesis might be responsible for the slow development of plant height and DBH in transgenic lines. This study provides an integrated analysis of the unintended effects of transgenic poplar, which will benefit the safety assessment and reasonable application of genetically modified trees.

## 1. Introduction

The introduction of foreign genes into the genome of the recipient plant enables the host plant to acquire novel traits. At the same time, the host plant may have unexpected and uncontrollable trait changes, which are called unintended effects [1]. It is necessary to assess the unintended effects of genetically modified (GM) plants to ensure they do not pose unacceptable risk [2]. Plants that introduce a single gene for insect resistance or herbicide resistance usually do not cause unintended effects [3]. Genetically engineered plants involved complex multigene networks, such as enhancing tolerance to abiotic stress, have a greater potential to introduce unintended effects [4].

Unintended effects can have either neutral or adverse impacts. In the study of GM herbicide-tolerant rice, it was discovered that, for most morphological or agronomic traits, there were no significant differences. In addition, some altered traits were not biologically relevant [5]. In *Petunia hybrida*, a *Clarkia breweri S*-linalool synthase cDNA (*lis*) was transferred and successfully expressed in all tissues, but linalool was not detected in nectaries, roots, pollen, and style [6]. However, in another study, the overexpression of a *Rosa rugosa* Thunb. *NUDX* gene in *P. hybrida* increased the contents of the main aroma components, while the blades of the transgenic petunias also became wider, and growth vigor became strong [7]. The underlying molecular mechanisms behind unintended effects remain unclear, although possible explanations include: (1) Transformation factors resulting from various stresses related to tissue handling, regeneration, and clonal propagation [8]. (2) Insertion effect that can arise when the integration site influences the transgenic plant due to the effect of the transgene on the functioning of surrounding sequences [9]. (3) Position effect arising from the influence of the integration site and transgene architecture on transgene expression level and stability [10]. (4) Induction effect that occurs when the expression product of foreign genes acts as an active factor to induce or silence the transcription and expression of endogenous genes [11]. Furthermore, it is worth noting that unintended effects are not unique to transgenic breeding, and traditional breeding methods such as hybridization and mutagenesis can also generate unintended effects [12].

Identification of insertion sites and flanking sequences of transferred DNA (T-DNA) is essential for the study of unintended effects. PCR, quantitative real-time PCR, and high performance liquid chromatography (HPLC) are often used to detect integration events and perform comparative analyses for the characterization of intended effects [13]. However, these detection methods have limitations since they may not cover all the unintended effects that transgenic plants may experience. In recent years, non-targeted approaches and omics technologies have provided new opportunities for T-DNA integration analysis [14]. The omics research strategy reduces the uncertainty in the evaluation of the integration event of transgenic plants and provides more in-depth information on the cell metabolism, which is considered a promising technology for the detection and evaluation of unintended effects [15].

Poplar is an economically important tree species worldwide, known for its fast growth and strong adaptability [16]. However, outbreaks of insect pests and soil salinization have hindered the development of the poplar industry. Genetic engineering has made progress in designing transgenic poplar with dual *Bacillus thuringiensis* (*Bt*) genes (*Cry1Ac* and *Cry3A*) to address insect pest issues [17]. Additionally, efforts have been made to develop insect-resistant and salt-tolerant transgenic poplar varieties by incorporating genes such as *BADH* and *NTHK1* [18]. While the transgenic poplar has brought considerable ecological benefits, it is important to consider the unintended effects of multigene cotransformation. Along with the positive benefits of multigene cotransformation in crops and trees, the unintended effects of T-DNA integration in this process should also be of concern. In this study, we selected three independent transgenic *PEN* lines carrying *Cry1Ac-Cry3A-BADH* genes and three independent lines carrying *Cry1Ac*-*Cry3A*-*NTHK1* genes in the field as research materials, and non-transgenic *PEN* was used as the control. We measured the growth of these transgenic lines to explore any unintended effects resulting from the multigene insertion. Furthermore, NGS resequencing was used to identify insertion sites and T-DNA insertion preferences. RNA-seq was then applied to elucidate the molecular mechanism underlying these unintended effects. The results provide important insights into the safety assessment of polygenic cotransformation and offer valuable information for breeding and managing new poplar varieties.

## 2. Results

### 2.1. Growth Traits of Transgenic Poplar in Field

**Figure 1:**
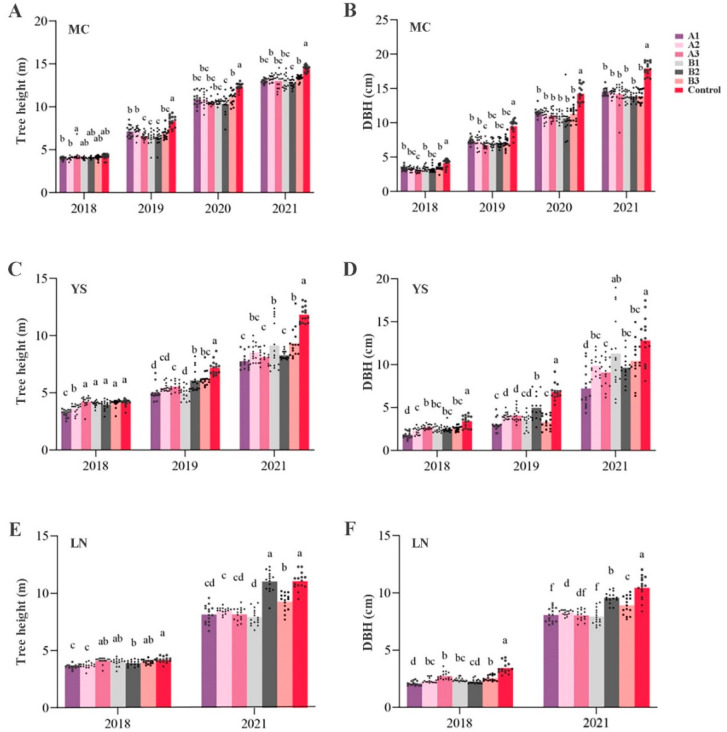
Tree height and diameter at breast height (DBH) of transgenic and non-transgenic *Populus* × *euramericana* cv. Neva (*PEN*) in three areas of Hebei province. (**A**) The tree height of transgenic and non-transgenic *PEN* in Mancheng (MC) area. (**B**) The DBH of transgenic and non-transgenic *PEN* in MC area. (**C**) The tree height of transgenic and non-transgenic *PEN* in Yanshan (YS) area. (**D**) The DBH of transgenic and non-transgenic *PEN* in YS area. (**E**) The tree height of transgenic and non-transgenic *PEN* in Luannan (LN) area. (**F**) The DBH of transgenic and non-transgenic *PEN* in LN area. Lowercase letters designate lines that significantly differ (*p* < 0.05).

To investigate the development of transgenic and non-transgenic lines, growth data from Mancheng (MC), Yanshan (YS), and Luannan (LN) were collected in different years (Figure 1). The tree height of MC demonstrated that the average tree height of transgenic lines in 2018 was 4.5% lower than that of the control, and in 2019, 2020, and 2021, the tree height of transgenic lines was significantly lower than that of the non-transgenic line, with average reductions of 18.5%, 13.3%, and 10%, respectively (Figure 1A). In the YS area, the average tree height of transgenic lines in 2018 was 8.7% lower than that of the control, and in 2019 and 2021, the tree height of transgenic lines was significantly lower, with average reductions of 23% and 30%, respectively (Figure 1C). Similarly, for LN, the average tree height of transgenic lines in 2018 and 2021 was 7.1% and 20% lower than that of the control, respectively (Figure 1E).

Regarding DBH, there were significant differences between transgenic and non-transgenic lines in MC for four consecutive years. The average DBH of transgenic lines was 20%, 25.2%, 22%, and 22% lower than that of the control (Figure 1B). In the YS area, the DBH of transgenic lines in 2018, 2019, and 2021 was significantly lower, with average reductions of 31%, 43.2%, and 25.2%, respectively (Figure 1D). In the LN area, the same trend was observed in 2018 and 2021, with the average DBH of transgenic lines being 30.1% and 35% lower than that of the control, respectively (Figure 1F).

### 2.2. Genotype by Environment Interaction Effect

To determine the environmental adaptability of transgenic and non-transgenic lines, we conducted variance analysis on the tree height and DBH across three regions in 2021. As shown in Table 1, there were no significant differences in tree height and DBH between blocks (*p* > 0.05), indicating that the sample replicates within the same region were consistent. However, significant variation in both tree height and DBH were observed among regions for the same line, as well as among different regions for the entire group of trees. This suggests a remarkable interaction effect between genotype and environment.

### 2.3. NGS Sequencing Quality Control and Data Comparison

After filtering the raw NGS sequencing data, we obtained ~15.8 G, ~19.1 G, ~17.4 G, ~15.8 G, ~19.1 G, and ~17.4 G of clean data for the transgenic lines A1, A2, A3, B1, B2, and B3, respectively. The sequencing depth was greater than 30X, and the Q30 value was greater than 90%, providing evidence of the sequencing quality and high data reliability (Supplementary Table S1). We detected seven insertion sites according to sequence alignment of the junction reads in all six samples (Table 2). All insertion sites were aligned to unilateral junction read.

### 2.4. PCR Verification of T-DNA Insertion Sites

We designed PCR primers based on the insertion site flanking sequence and T-DNA boundary information to verify the NGS sequencing results (Supplementary Table S2). The PCR results showed that only four insertion sites in the A2, A3, and B3 transgenic lines were validated (Table 2), with slight differences in product sizes from predicted bands. The PCR-amplified products were then sequenced and aligned to the genome and vector sequences, and the results proved the accuracy of the four insertion sites detected by NGS sequencing. The alignment results were shown in Supplementary Table S3 and Figure S1. The boundary sequence on the vector was moderately damaged, and some small fragments of the genome were deleted.

### 2.5. T-DNA Insertion Location and Adjacent Genes

Among the seven insertion sites of the six transgenic lines, three were located in the gene region and four were located in the intergenic region (Supplementary Table S4). Three insertion sites were located in the following gene regions: the first exon of the *LOC7498022* gene on chromosome 10 of B1, the first intron of the *LOC18104831* gene on chromosome 14 of B2, and the second exon of the *LOC18100061* gene on chromosome six of B3. A total of 21 genes were found within 20 kb upstream and downstream of insertion sites, including 19 protein-coding genes and two non-coding genes, such as abscisic acid 8’-hydroxylase 2, growth-regulating factor 4. In the 1000 bp ranges extending from upstream and downstream of each insertion site, the AT base contents of A1, A2, A3, B1, B2, and B3 were 67.4%, 47.4%, 75.8%, 57.8%, 67.2%, 64.2%, and 69.7%, respectively. The AT base content in the adjacent insertion sites of A1, A3, B2, and chromosome 18 in B3 was higher than the average AT content in chromosomes.

### 2.6. RNA-seq and Differentially Expressed Genes (DEGs) Analysis

**Figure 2:**
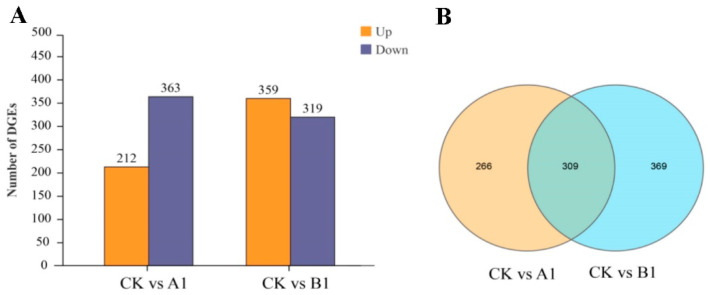
Number of differentially expressed genes (DEGs). (**A**) The number of differentially expressed genes (DEGs) in A1 and B1 lines. (**B**) Venn diagram of DEGs in A1 and B1 lines.

After filtering out adapter and low-quality reads from the raw data, 55.85 G clean bases were obtained from nine samples. The mapping rate of reads among the samples was > 86%, with an average GC content of 44.28% and Q30 > 93%, indicating good sequencing quality (Supplementary Table S5). A total of 31640 genes were identified in the genome of *P. trichocarpa*. In addition, some reads were identified in the non-gene region of the reference genome, which were re-annotated as new genes. A total of 1081 new genes were predicted, of which 1009 genes were functionally annotated. Compared with the control, 575 DEGs (212 upregulated and 363 downregulated) were identified in A1 line and 678 DEGs (359 upregulated and 319 downregulated) were identified in B1 line (Figure 2A). In the A1 and B1 lines there were 309 common DEGs, and 266 and 369 specific DEGs, respectively. Common DEGs accounted for 32.73% (Figure 2B).

### 2.7. Gene Ontology (GO) Enrichment Analysis

**Figure 3:**
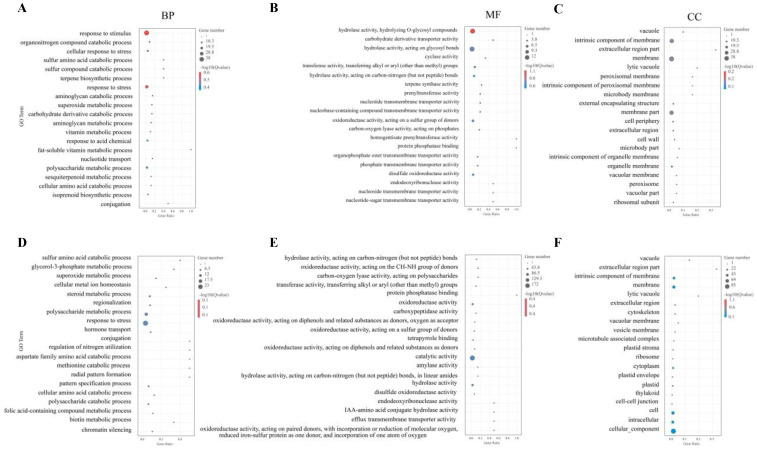
GO terms enrichment. (**A**–**C**) The top 20 significantly enriched GO terms of DEGs in biological processes (BP), molecular function (MF), and cellular component (CC) of A1 transgenic line. (**D**–**F**) The top 20 significantly enriched GO terms of DEGs in BP, MF, and CC of B1 transgenic line.

The GO enrichment terms of DEGs in the A1 and B1 lines showed similar results, with the most DEGs involved in biological processes, followed by molecular function, and the fewest in the cellular component (Supplementary Figure S2). In biological processes, the DEGs were mainly enriched in metabolic processes, cellular processes, and single-organism process. Concerning the molecular function, the DEGs were mainly enriched in catalytic activity and binding. In terms of the cellular component, the DEGs were mainly enriched in the cell, cell part, and membrane. As shown in Figure 3, the biological processes (BP) involving DEGs in the A1 transgenic line were significantly enriched in terms of “response to stimulus”, “organonitrogen compound catabolic process”, “response to stress”. The molecular function (MF) GO terms related to “hydrolase activity, hydrolyzing O-glycosyl compounds”, “carbohydrate derivative transporter activity”, “hydrolase activity, acting on glycosyl bond” were significantly enriched. Regarding the cellular component (CC), GO terms related to “vacuole”, “intrinsic component of membrane”, “extracellular region part” were significantly enriched. Similarly, the DEGs in the B1 transgenic line were significantly enriched in terms of “sulfur amino acid catabolic process”, “glycerol-3-phosphate metabolic process”, and “cellular metal ion homeostasis” in BP. “Protein phosphatase binding” GO terms were notably enriched in MF. Additionally, GO terms related to “vacuole”, “extracellular region part”, and “lytic vacuole” were significantly enriched in CC.

### 2.8. Kyoto Encyclopedia of Genes and Genomes (KEGG) Metabolic Pathway Analysis

**Figure 4:**
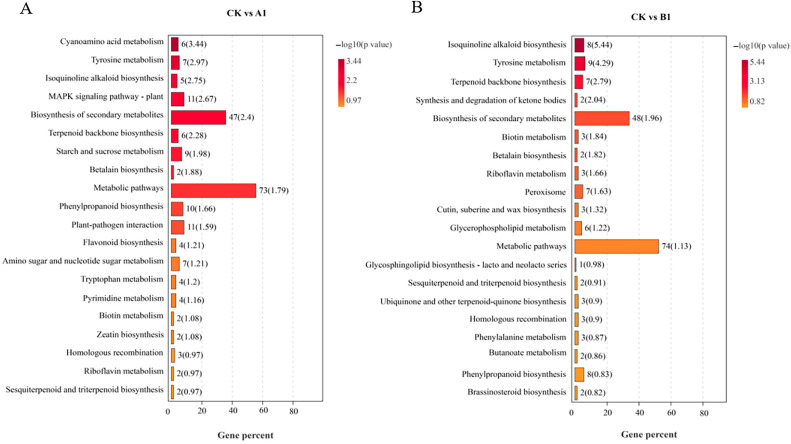
The top 20 significantly enriched KEGG pathways of DEGs. (**A**) KEGG enrichment pathways of DEGs in A1 line. (**B**) KEGG enrichment pathways of DEGs in B1 line.

KEGG pathway analysis was used to investigate the unintended effects of transgenic lines. The significantly enriched pathways are shown in Figure 4. Out of 131 DGEs from the A1 line, 90 pathways were found to be enriched. The top 20 pathways with the highest significance include “Cyanoamino acid metabolism”, “Tyrosine metabolism”, “Isoquinoline alkaloid biosynthesis”, etc. A total of 141 DGEs from the B1 line were enriched in 84 metabolic pathways. The top 20 significantly enriched pathways consist of “Isoquinoline alkaloid biosynthesis”, “Tyrosine metabolism”, “Terpenoid backbone biosynthesis”, etc. A total of 68 pathways showed co-enrichment of DEGs from both the A1 and B1 lines, including “plant hormone transduction”, “Phenylpropanoid biosynthesis”, “Zeatin biosynthesis”, “MAPK signaling pathway”, and other important pathways related to plant growth and development.

### 2.9. K-Means Clustering of DEGs Expression Patterns

**Figure 5:**
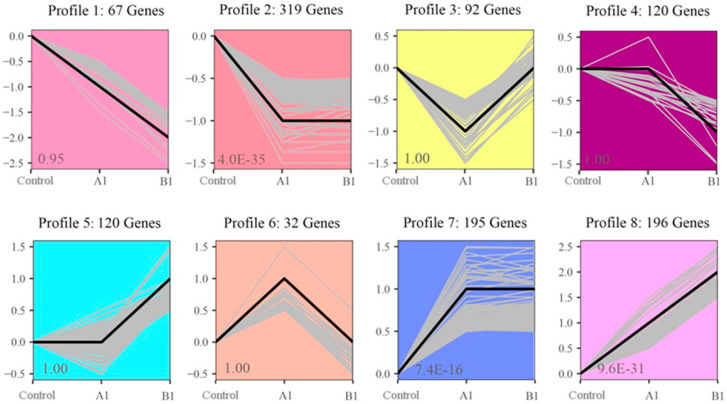
Hierarchical cluster analysis of differentially expressed genes (DEGs).

To further understand the gene expression patterns of transgenic lines, we performed a K-means cluster analysis of the DEGs (Figure 5). All the DEGs were clustered into eight expression patterns. Among these clusters, Profiles 2, 7, and 8 contained the most genes and showed significant correlation. The expression patterns of the A1 and B1 lines in Profiles 2 and 7 were consistent. Profile 2 contained 319 genes, and the gene expression levels of A1 and B1 were significantly down-regulated. Profiles 7 and 8 contained 195 and 196 genes, respectively; the gene expressions of A1 and B1 were significantly up-regulated compared to control.

### 2.10. Analysis of Genes Expression Related to Growth and Development

**Figure 6:**
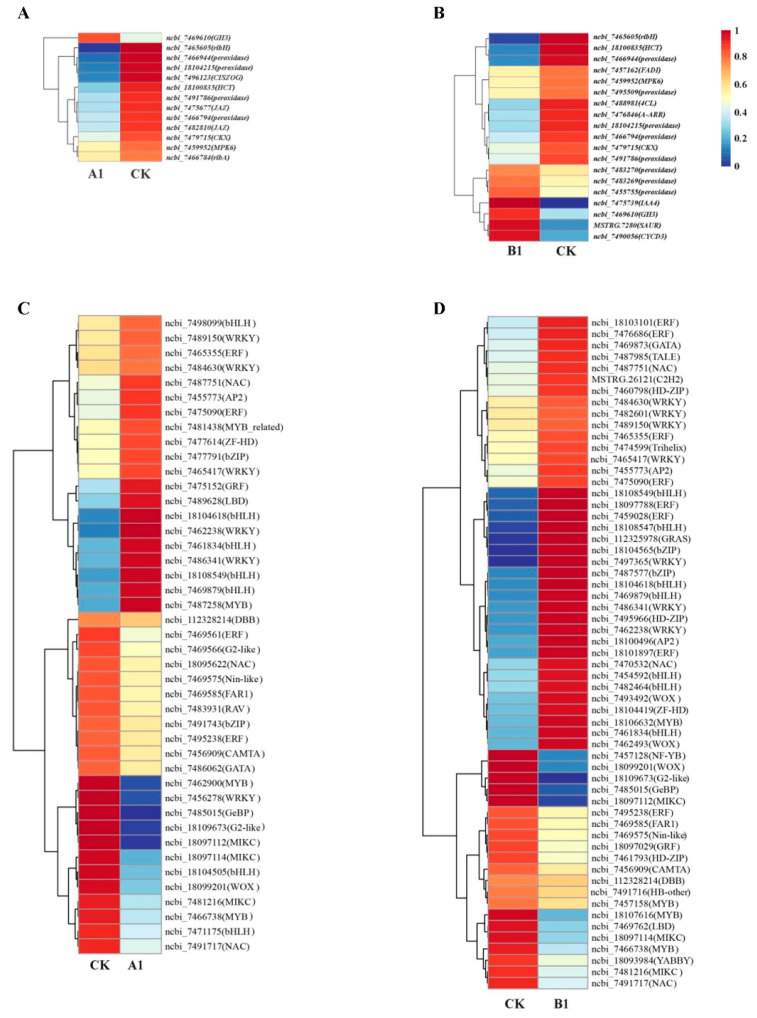
Heat map of growth-related genes and transcription factors (TFs). (**A**,**B**) Expression levels of differentially expressed genes (DEG) related to growth and development in the A1 (**A**) and B1 (**B**) transgenic lines. (**C**,**D**) Expression levels of differentially expressed TFs in A1 (**C**) and B1 (**D**).

To determine the influence of exogenous gene insertion on plant growth and development, we analyzed the expression levels of genes related to growth in the A1 and BI transgenic lines (Figure 6A,B). The gene expression revealed that two *JAZ* genes and *MPK6* gene of the A1 transgenic line were significantly down-regulated in the plant hormone transduction pathway, while *GH3* was significantly up-regulated. *CKX* and *CISZOG* genes that are involved in the zeatin biosynthesis pathway were significantly down-regulated in A1 line (Figure 6A). Likewise, in the B1 transgenic line, the *CKX* gene was significantly down-regulated in the zeatin biosynthesis pathway (Figure 6B). Moreover, the *MPK6*, *PP2C*, and *SnRK* genes were down-regulated while the *SAUR*, *AUX/IAA*, *GH3*, and *CYCD3* genes were up-regulated in the plant hormone transduction pathway of the B1 transgenic line (Figure 6B). In the phenylpropanoid biosynthesis pathway, the *HCT* and *Peroxidase* genes involved in lignin synthesis were significantly down-regulated in both transgenic lines, and *4CL* gene was significantly down-regulated in the B1 transgenic line.

### 2.11. Analysis of Differentially Expressed Transcription Factors (TFs)

TFs play a vital role in regulating plant growth and enhancing stress resistance. In this study, a total of 2359 TFs were screened, and 76 TFs showed a significant difference in expression across different comparison groups. These TFs were classified into 29 families, with the most abundant ones being bHLH, WRKY, ERF, MYB, and NAC. In the A1 transgenic line, 43 TFs exhibited significant expression changes, with 20 up-regulated and 23 down-regulated genes (Figure 6C). These genes belong to 21 TF families. The bHLH and WRKY families had the largest number of genes, and most of them were predominantly up-regulated. Regarding the B1 line, the expression levels of 59 TFs were significantly altered, with 43 up-regulated and 16 down-regulated (Figure 6D). These genes belonged to 27 TF families, and the ERF, WRKY, and bHLH families had the largest number. Most DGEs in the ERF family showed up-regulation, while all DGEs in the bHLH and WRKY families were predominantly up-regulated.

### 2.12. Verification of RNA-seq Data by Quantitative Reverse-Transcription PCR (qRT-PCR)

**Figure 7:**
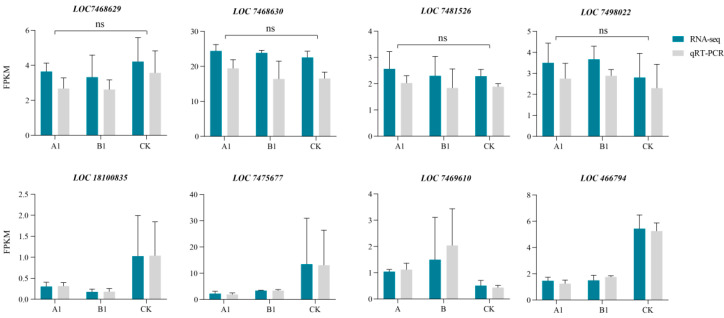
qRT-PCR validation of the eight genes in two transgenic lines. *LOC7468629*, *LOC7468630*, *LOC7481526*, and *LOC7498022* were four genes located within 20 kb of the T-DNA insertion site; *LOC18100835*, *LOC7475677*, *LOC7469610*, and *LOC466794* were four genes commonly expressed in the A1 and B1 lines.

We used qRT-PCR to measure the expression level of selected genes. We focused on four genes that were commonly expressed in the A1 and B1 lines (*LOC18100835*, *LOC7475677*, *LOC7469610*, *LOC466794*), as well as four genes located within 20 kb of the T-DNA insertion site (*LOC7468629*, *LOC7468630*, *LOC7481526*, *LOC7498022*). The four protein-coding genes near the insertion site were all effectively expressed (FPKM > 1), and the gene expression levels of A1 and B1 lines were not significantly different from those of the control (Figure 7). Real-time PCR revealed the same tendency in changes in expression as the RNA-seq data, which suggests that the RNA-seq data in this study are reliable.

## 3. Discussion

Transgenic plants are labeled with exogenous T-DNA, making it essential to identify their unintended effects for commercial promotion and risk assessment. In *Arabidopsis*, engineering stress-tolerance genes did not cause unintended effects [19]. However, transgenic rice expressing *Bt* toxin showed that the *Bt* protein had unintended effects on non-target invertebrates [20]. Our previous studies demonstrated that *Bt* transgenic poplar had no significant effect on the arthropod community or bacterial diversity, nor did it impact tree height. However, it did affect the radial development of the receptor [21,22]. In this study, the transgenic *PEN* carrying the *Cry1Ac*-*Cry3A*-*BADH* genes and the *Cry1Ac*-*Cry3A*-*NTHK1* genes had significantly inhibited tree heights and DBH at different growing sites (Figure 1). Therefore, analyzing unintended effects of transgenic plants case by case is necessary.

### 3.1. T-DNA Integration Analysis

Exogenous gene integration usually occurs randomly on the chromosome of the recipient genome. Choi et al. (2002) used fluorescence in situ hybridization (FISH) to analyze transgenic plants, and the results showed that there were no preferential integration sites of foreign DNA among chromosomes [23]. This study also found no obvious chromosomal bias in T-DNA insertion, which is consistent with previous findings. However, it is worth mentioning that the sample size in this study was small, and further data from transgenic lines are needed to explore whether T-DNA integration on chromosomes is biased or not. It has been recognized that the expression level of exogenous genes will vary depending on their randomly inserted positions [24]. Generally, the expression of exogenous genes is unstable when T-DNA is integrated near the heterochromatin region, while it is stable when T-DNA is integrated into the euchromatin region with high transcriptional activity [25,26]. In the genome, T-DNA is more likely to insert into intergenic regions rather than gene regions [27]. In this study, three insertion sites were located in the gene region and four were located in the intergenic region (Supplementary Table S4). Furthermore, transgenic lines with *Agrobacterium*-mediated integration usually have a relatively small copy number [28]. In this study, six transgenic lines of *PEN* were all generated using the *Agrobacterium*-mediated method, resulting in a total of seven T-DNA insertion sites, all of which were single-copy integrations (Table 2). The integrated form of a single copy or limited number of copies may be more conducive to the expression of exogenous genes [29]. Previous studies have shown that T-DNA tends to integrate into AT-rich regions and intergenic regions [30]. According to the analysis of 1000 bp sequences near the seven loci in this study, the AT content of four loci was higher than the average of the chromosomes.

In previous studies, the analysis of exogenous gene integration sites and flanking sequences has been mostly based on PCR and other similar methods. T-DNA flanking sequences have been successfully identified in a variety of crops [31]. However, these methods are complex, time consuming, possess poor specificity, and are unable to discover all sites of insertion and flanking sequences of multilocus insertion. The NGS technology can overcome the limitations of PCR-based methods and provide rapid and comprehensive molecular characterization data. The quality of sequencing library construction and sequencing depth are two pivotal factors that affect NGS sequencing results [32]. In this study, utilizing NGS re-sequencing technology, 129.72 G of sequencing data was obtained, and six insertion sites of transgenic *PEN* trees were identified. Four of these were subsequently verified by PCR and Sanger sequencing (Table 2).

The integration of T-DNA into the recipient genome often leads to duplication, deletion, and inversion of T-DNA or the recipient genome DNA [33]. In this study, the flanking sequence of the genome near the T-DNA integration site was also partially deleted compared with the reference genome (Supplementary Table S3). The reason may be the loss of fragments, or there may be differences between the genome of *PEN* and the reference genome of *P. trichocarpa*. Some insertion sites in this study could not be verified by PCR amplification, which may be caused by false positives in the comparison of NGS data [34,35]. On the other hand, the left and right boundary sequences of the vector used in this study are short. If PCR primers are located in the boundary sequence, the product may not be obtained due to partial loss of the boundary sequence during integration.

### 3.2. T-DNA Integration and Gene Expression

Gene stacking biotechnology is a way to combine multiple traits in plants. Nowadays, crops with gene stacking have been planted in many countries; however, the combination of multiple genes may also lead to mutual interference between genes, causing one gene to inhibit the other gene. According to previous research, a comparison and analysis of the expression of exogenous genes in four double *Bt* gene vectors, showed that the expression level of the *Bt* gene was low when it was located upstream of T-DNA [36]. With the development of transgenic technology, it is necessary to note whether gene stacking transgenic plants are safer than single gene transgenic plants. Some studies suggested that breeding by stacking two transgene events does not introduce greater variation than conventional breeding processes [37,38]. Proteomic profiling analysis found no newly expressed proteins in transgenic maize (12-5 × IE034) with insect-resistant and glyphosate-tolerant genes. The differences in protein expression between transgenic plants and their parents were also within the range of variation of conventional maize varieties. This suggests that stacked-trait development via conventional breeding did not have an impact on the genetic stability of T-DNA [39]. Transgenic poplar trees with a single *Bt* gene have been shown to either improve yields or affect DBH development [22,40].

In this study, the results indicated that insect-resistant and salt-tolerant gene stacking further inhibited the growth of poplar trees. The growth rate of DBH and tree height were significantly lower in transgenic lines than in non-transgenic lines. This may be caused by both exogenous promoter overexpression and gene stacking. A study has shown that, when the transgenic line uses a constitutive promoter (CaMV 35S), the transgenic rice plants exhibit a dwarfing phenotype under normal growth conditions [41]. The CaMV 35S promoter can drive high gene expression even when plants do not require it, which may disrupt normal gene regulation, metabolic pathways, and affect overall growth and development. Gene stacking also intensifies the accumulation of foreign genes in the receptor, and the interaction between exogenous genes may bring “byproducts” that affect growth and development.

The growth and development of transgenic poplar are closely related to gene transcription regulation. RNA-seq technology can analyze the molecular mechanism of inhibiting the growth of DBH and tree height of transgenic PEN to some extent [42]. In this study, 575 and 678 DEGs, including bHLH, WRKY, ERF, MYB, NAC, and other TFs, were screened in the A1 and B1 transgenic lines by RNA-seq (Figure 2). There is a positive correlation that exists between lignin content and DBH development, and transcriptional regulation is the main mechanism of gene expression related to lignin biosynthesis [43]. The NAC and bHLH TF families can regulate lignin synthesis, while the MYB family plays a key role in lignin synthesis [44,45]. The WRKY TF family plays an indispensable role in the formation and development of plant roots, stems, and leaves, and some WRKY TFs can regulate diameter elongation [46,47]. Among the pathways of DEG enrichment, the A1 and B1 lines were co-enriched in important pathways related to plant growth and development, such as plant hormone transduction, phenylpropanoid biosynthesis, and zeatin biosynthesis. Some auxin early response genes (*SAUR*, *IAA4*, *GH3*) and *CYCD3* of the A1 or B1 line were significantly up-regulated, while cytokinin dehydrogenase (*CKX*), jasmonic acid response inhibitor gene (*JAZ*), and key genes in the lignin pathway (*4CL*, *HCT*) and enzymes were significantly down-regulated (Figure 6A,B).

Most of the early auxin response genes belong to the family of *Aux/IAAs*, *GH3s*, and *SAURs* [48]. The *GH3* gene is associated with plant growth and development [49]. In *A. thaliana*, the gene product of *GH3* is involved in auxin signal transduction and inhibits shoot and hypocotyl cell elongation and lateral root cell differentiation [50]. Overexpression of *CYCD3* in *A. thaliana* leads to a radical alteration in leaf architecture and inhibiting differentiation [51]. *CKX* has been shown to be involved in the regulation of cytokinin homeostasis and plays a key regulatory role in plant growth and development [52]. Both 4CL and HCT are key enzymes in the phenylpropane metabolism pathway, and inhibiting 4CL activity in tobacco leads to a decrease in lignin content [53]. In summary, changes in the transcriptional expression of some TFs and genes related to plant growth and lignin synthesis may be the main reason for the growth inhibition of transgenic *PEN* in this study.

## 4. Conclusions

In this study, successive years of experimental tests were conducted at different sites to investigate the unintended effects of exogenous T-DNA integration in poplar. The obtained results provide theoretical support for the application of transgenic trees in the field. Based on the data, T-DNA integration events carrying multiple genes led to the inhibition of tree height and DBH in *PEN*. NGS and transcriptome sequencing analysis revealed that the growth inhibition of transgenic *PEN* may be attributed to the influence of exogenous genes on the expression of genes related to plant hormone transduction and lignin synthesis, rather than the damage done by T-DNA to the receptor genome and its influence on adjacent genes. In conclusion, all the experimental data in this study were obtained from plants in the field, and it was found that there were unintended effects in multigene cotransformation. In the study of transgenic plants, especially to investigate the unintended effects, field testing is necessary, which can provide results that may not be obtained in laboratory conditions. Additionally, the results of this study remind us to pay special attention to the growth inhibition caused by multigene cotransformation and to address this issue in the research of woody plant transgenic for wood or biomass harvesting.

## 5. Materials and Methods

### 5.1. Plant Materials and Study Sites

**Figure 8:**
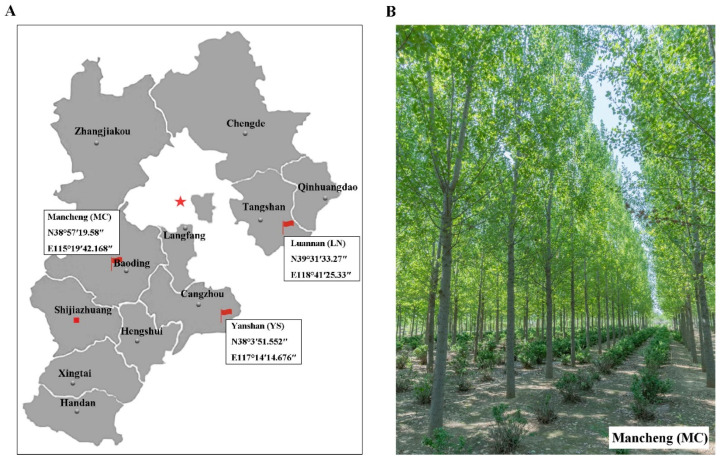
Location of transgenic *Populus* × *euramericana* cv. Neva (*PEN*) forest in three areas of Hebei province. (**A**) Location of transgenic *PEN* forests. (**B**) Experimental forest in Mancheng (MC) area.

Three transgenic lines of *PEN* carrying the *Cry1Ac*-*Cry3A*-*BADH* genes (A1–A3) and three transgenic lines of *PEN* carrying the *Cry1Ac*-*Cry3A*-*NTHK1* genes (B1–B3) were obtained in our laboratory by *Agrobacterium*-mediated genetic transformation [18,54]. These lines have been approved by the State Forestry and Grassland Bureau for environmental release. Non-transgenic *PEN* was used as the control. On April 2018, a random block design with three replicates was employed to construct field trial forest in MC District, Baoding City, Hebei Province (E115°19′ and N38°57′), LN, Tangshan City, Hebei Province (E118°41′ and N39°31′), and YS, Cangzhou City, Hebei Province (E117°14′ and N38°3′) (Figure 8). Each plot contained nine clones, with a plant spacing of 2 m and row spacing of 4 m. Natural conditions and management practices were consistent across the test sites.

### 5.2. Growth Characteristics

Between 2018 and 2021, the plant height and DBH of six transgenic and control lines at three locations were measured in November. Based on the high consistency of data collected from different locations in 2018 and the impact of COVID-19 in 2020, we collected growth data for four consecutive years (2018–2021) as typical representative in MC. We also collected data from YS in 2018, 2019, and 2021, as well as data from LN in 2018 and 2021. Measurements were taken from 27 trees per line in three plots. The diameter of the trunk at 1.3 m from the ground was measured to determine the DBH. A Bruce altimeter was used to measure the plant height.

### 5.3. NGS Resequencing

The young leaves of six transgenic lines were collected for NGS sequencing. The DNA of the samples was broken into fragments of about 300 bp by ultrasound, which fragments were purified, and the ends were repaired. Subsequently, the 3’ end was added to A and the sequencing adaptor was connected. The appropriate size of fragments was chosen through agarose gel electrophoresis. Then, the fragments were amplified by PCR to construct sequencing libraries. After quality control, the libraries were sequencing by the Illumina HiSeq (San Diego, CA, USA) platform at the Beijing BIOMICS Co., Ltd. Quality control was performed on the obtained raw data to remove reads containing adaptor and ones of low quality. Clean reads were mapped to the genome of *P. trichocarpa* and vector sequences through the BWA algorithm of the AIM-HII software [55], in order to search for exogenous insertions. The junction reads that were aligned to both the genome and T-DNA sequences were selected, and the position and direction of insertion were determined according to the alignment information of junction reads.

### 5.4. T-DNA Integration Verification and Flanking Sequence Analysis

The PCR primers were designed based on the genomic sequences within 600 bp upstream and downstream of the T-DNA insertion sites (Supplementary Table S2). T-DNA sequence information, which was obtained through NGS resequencing, was also taken into account. The DNA of six transgenic lines was amplified by PCR to verify the results of NGS resequencing. The total PCR amplification volume was 40 μL including: 20 μL 2 × M5 Hiper plus Taq HiFi PCR mix (with blue dye), 2 μL DNA, 1 μL each of forward and reverse primers, and 16 μL deionized water. The amplification procedure was: pre-denaturation at 95 °C for 5 min, followed by denaturation at 94 °C for 50 s, annealing at 55–58 °C for 30 s, and extension at 72 °C for 1 min for 30 cycles, and after cycles were completed, hold for 5 min at 72 °C. The PCR products were sequenced by first-generation Sanger at BGI Genomics (Shenzhen, China). The sequences were aligned to the genome and vector sequence to identify the integrity of the T-DNA integration sequence and flanking sequence of the insertion sites. The adjacent genes within 20 kb upstream and downstream of each insertion site were identified and their functions analyzed according to the genome annotation. The AT content within 1000 bp upstream and downstream of the insertion site was also analyzed statistically.

### 5.5. RNA-seq and Data Quality Control

In May 2019, the third fully expanded leaves from top to bottom from the same part of A1, B1, and control lines were collected in the MC experimental forest for transcriptome sequencing, with three biological replicates per line. The leaves were immediately frozen in liquid nitrogen after collection. Total RNA extraction was performed in the laboratory using the plant total RNA extraction kit (Takara, Dalian, China), following the manufacturer’s instructions. RNA quality testing and library construction, quality control, and sequencing were conducted at Gene Denovo Biotechnology Co., Ltd. (Guangzhou, China) using the Illumina HiSeq 2500 platform. Clean reads were obtained after quality control and low-quality data filtering. Reads containing adapters, an N ratio greater than 10%, and all A-bases were removed. The clean data were then used for sequence comparison through Hisat2 v2.0.1.

### 5.6. DEGs Identification and Functional Analysis

Gene expression levels were measured based on fragments per kilobase of transcript per million base pairs sequenced (FPKM), and DEGs were identified using the DESeq 2 R package (1.10.1) [56]. The thresholds for DEGs were set to fold change |log2FC| ≥ 1 and significance testing (*p* < 0.05). GO analysis and the KEGG database were used to examine the functional enrichment of DEGs at a significance level of *p* < 0.05. K-means hierarchical clustering was used to analyze gene expression trends.

### 5.7. qRT-PCR

Eight genes were performed using qRT-PCR and *Actin* as an internal reference to verify the RNA-seq results. First-strand complementary cDNA synthesis for gene quantification was carried out using the First-Strand cDNA synthesis Kit (TranGen, Beijing, China). Gene expression was quantified by SYBR green qPCR Master Mix (Tiangen, Beijing, China) according to the manufacturer’s instructions. Three biological and three technical replicates were performed for each transgenic line, and the relative expression levels were calculated using the 2^–ΔΔCt^ method. The primer sequences are listed in the Supplementary Table S2.

### 5.8. Statistical Analysis Methods

Microsoft Excel 2016 was used to create tables. IBM SPSS statistics v26.0 software was used to conduct one-way analysis of variance (ANOVA) and Duncan’s multiple range test (*p* < 0.05). The DPS v19.05 software was used to test the significance of the interaction effect between transgenic lines and the environment (*p* < 0.05). Graphpad Prism v9.3, as well as R v4.3.1 software, was used for data visualization and analysis.

### 5.9. Availability of Data and Material

The datasets generated and/or analyzed during the current study have been deposited in the NCBI Sequence Read Archive repository under accession numbers PRJNA995219 and PRJNA995045 (https://www.ncbi.nlm.nih.gov/bioproject, accessed on 15 August 2023). Any reasonable requests should be directed to the corresponding author.

## Figures and Tables

**Table 1:** Variance analysis of transgenic and non-transgenic lines for three regions in 2021.

Growth Trait	Type of Variation	Degree of Freedom	Sum of Squares	MEAN SQUARE	*F*	*P*
Tree height	Block	6	0.4136	0.0689	0.34	0.9126
	Region	2	251.855	125.9275	616.22	0.0001
	Line	6	55.7341	9.289	45.46	0.0001
	Line × site	12	24.447	2.0372	9.97	0.0001
	Error	36	7.3568	0.2044	-	-
	Total	62	339.8065	-	-	-
DBH	Block	6	0.6221	0.1037	0.1562	0.9865
	Region	2	289.2402	144.6201	217.8217	0.0001
	Line	6	171.7252	28.6209	43.1077	0.0001
	Line × site	12	60.1608	5.0134	7.551	0.0001
	Error	36	23.9018	0.6639	-	-
	Total	62	545.65	-	-	-

**Table 2:** Location information and detection results of T-DNA insertion sites.

Exogenous Genes	Chromosome	Transgenic Lines	Insertion Position	Insert Direction	Reads	Verified Subsequently
*Cry1Ac*-*Cry3A*-*BADH*	Chr09	A1	4,383,212–4,383,306	Reverse	11	-
	Chr14	A2	10,334,700–10,334,833	Reverse	4	Yes (single-ended)
	Chr13	A3	16,011,211–16,011,348	Forward	12	Yes (single-ended)
*Cry1Ac*-*Cry3A*-*NTHK1*	Chr10	B1	10,079,143–10,079,241	Reverse	6	-
	Chr14	B2	1,944,472–1,944,710	Forward	12	-
	Chr06	B3	9,014,954–9,015,182	Forward	12	Yes (single-ended)
	Chr18		15,956,346–1,595,632	Reverse	9	Yes (single-ended)

T-DNA insertion information was searched with the assistance of *Agrobacterium* Insertional Mutagenesis High-throughput Insert Identification (AIM-HII) software. In the table, “Chromosome” and “Insertion Position” represent the chromosome and position detected at the T-DNA insertion site. “Insert Direction” indicates the insertion direction of T-DNA determined after reading sequence alignment; “Reads” refers to the number of fragments containing both T-DNA sequence and genome sequence information recognized by AIM-HI software; “Verified Subsequently” means subsequent verification by PCR.

## Data Availability

Data supporting reported results can be found after 15 August 2023.

## Supplementary Materials

The following supporting information can be downloaded at: https://www.mdpi.com/article/10.3390/ijms241612952/s1.

## References

[1] Wang Y., Liu Q., Song X., Yang X., Han L., Romeis J., Li Y. (2022). Unintended changes in transgenic maize cause no nontarget effects. Plants People Planet.

[2] Li Y., Zhang Q., Liu Q., Meissle M., Yang Y., Wang Y., Hua H., Chen X., Peng Y., Romeis J. (2017). *Bt* rice in China—Focusing the nontarget risk assessment. Plant Biotechnol. J..

[3] Liu Q., Yang X., Tzin V., Peng Y., Romeis J., Li Y. (2020). Plant breeding involving genetic engineering does not result in unacceptable unintended effects in rice relative to conventional cross-breeding. Plant J..

[4] Jiang Q., Niu F., Sun X., Hu Z., Li X., Ma Y., Zhang H. (2017). RNA-seq analysis of unintended effects in transgenic wheat overexpressing the transcription factor *GmDREB1*. Crop J..

[5] Jiang X., Xiao G. (2010). Detection of Unintended Effects in Genetically Modified Herbicide-tolerant (GMHT) Rice in Comparison with Non-target Phenotypic Characteristics. Afr. J. Agric. Res..

[6] Lucker J., Bouwmeester H.J., Schwab W., Blaas J., van der Plas L.H., Verhoeven H.A. (2001). Expression of *Clarkia S*-linalool synthase in transgenic petunia plants results in the accumulation of *S*-linalyl-beta-D-glucopyranoside. Plant J..

[7] Sheng L., Zang S., Wang J., Wei T., Xu Y., Feng L. (2021). Overexpression of a *Rosa rugosa* Thunb. NUDX gene enhances biosynthesis of scent volatiles in petunia. PeerJ.

[8] Filipecki M., Malepszy S. (2006). Unintended consequences of plant transformation: A molecular insight. J. Appl. Genet..

[9] Ouakfaoui S.E., Miki B. (2005). The stability of the *Arabidopsis* transcriptome in transgenic plants expressing the marker genes *nptII* and *uidA*. Plant J..

[10] Matzke A.J., Matzke M.A. (1998). Position effects and epigenetic silencing of plant transgenes. Curr. Opin. Plant Biol..

[11] Stam M., Mol J.N.M., Kooter J.M. (1997). Review article: The silence of genes in transgenic plants. Ann. Bot..

[12] Herman R.A., Price W.D. (2013). Unintended compositional changes in genetically modified (GM) crops: 20 years of research. J. Agric. Food Chem..

[13] Metzdorff S.B., Kok E.J., Knuthsen P., Pedersen J. (2006). Evaluation of a non-targeted “Omic” approach in the safety assessment of genetically modified plants. Plant Biol..

[14] Davies H. (2010). A role for “omics” technologies in food safety assessment. Food Control.

[15] Kumar R., Bohra A., Pandey A.K., Pandey M.K., Kumar A. (2017). Metabolomics for plant improvement: Status and prospects. Front. Plant Sci..

[16] Biselli C., Vietto L., Rosso L., Cattivelli L., Nervo G., Fricano A. (2022). Advanced breeding for biotic stress resistance in poplar. Plants.

[17] Ren Y., Zhou X., Dong Y., Zhang J., Wang J., Yang M. (2021). Exogenous gene expression and insect resistance in dual Bt toxin *Populus* × *euramericana* ‘Neva’ transgenic plants. Front. Plant Sci..

[18] Yang R.L., Wang A.X., Zhang J., Dong Y., Yang M.S., Wang J.M. (2016). Genetic transformation and expression of transgenic lines of *Populus* x *euramericana* with insect-resistance and salt-tolerance genes. Genet. Mol. Res..

[19] Abdeen A., Schnell J., Miki B. (2010). Transcriptome analysis reveals absence of unintended effects in drought-tolerant transgenic plants overexpressing the transcription factor *ABF3*. BMC Genom..

[20] Yang H., Peng Y., Tian J., Wang J., Hu J., Song Q., Wang Z. (2017). Review: Biosafety assessment of *Bt* rice and other *Bt* crops using spiders as example for non-target arthropods in China. Plant Cell Rep..

[21] Zuo L., Yang R., Zhen Z., Liu J., Huang L., Yang M. (2018). A 5-year field study showed no apparent effect of the *Bt* transgenic 741 poplar on the arthropod community and soil bacterial diversity. Sci. Rep..

[22] Huang Y., Zhen Z., Cui Z., Liu J., Wang S., Yang M., Wu J. (2021). Growth and arthropod community characteristics of transgenic poplar 741 in an experimental forest. Ind. Crops Prod..

[23] Choi H., Lemaux P., Cho M.J. (2002). Use of fluorescence in situ hybridization for gross mapping of transgenes and screening for homozygous plants in transgenic barley (*Hordeum vulgare* L.). Theor. Appl. Genet..

[24] Wang Y., Yang Y., Wang F., Wang G., Wang C., Wang W., Chen K., Gu C., Yu Q., Jiang J. (2021). Growth adaptability and foreign gene stability of *TaLEA* transgenic *Populus simonii* × *nigra*. Ann. For. Sci..

[25] Francis K.E., Spiker S. (2005). Identification of *Arabidopsis thaliana* transformants without selection reveals a high occurrence of silenced T-DNA integrations. Plant J..

[26] Kim S., Gelvin S.B. (2007). Genome-wide analysis of *Agrobacterium* T-DNA integration sites in the *Arabidopsis* genome generated under non-selective conditions. Plant J..

[27] Li Y., Rosso M.G., Ülker B., Weisshaar B. (2006). Analysis of T-DNA insertion site distribution patterns in *Arabidopsis thaliana* reveals special features of genes without insertions. Genomics.

[28] Kharb P., Chaudhary R., Tuteja N., Kaushik P. (2022). A genotype-independent, simple, effective and efficient in planta *Agrobacterium*-mediated genetic transformation protocol. Methods Protoc..

[29] Travella S., Ross S.M., Harden J., Everett C., Snape J.W., Harwood W.A. (2005). A comparison of transgenic barley lines produced by particle bombardment and *Agrobacterium*-mediated techniques. Plant Cell Rep..

[30] Zhou X., Ren Y., Wang S., Chen X., Zhang C., Yang M., Dong Y. (2022). T-DNA integration and its effect on gene expression in dual *Bt* gene transgenic *Populus* × *euramericana* cv. Neva. Ind. Crops Prod..

[31] Yang L., Xu S., Pan A., Yin C., Zhang K., Wang Z., Zhou Z., Zhang D. (2005). Event specific qualitative and quantitative polymerase chain reaction detection of genetically modified MON863 maize based on the 5′-transgene integration sequence. J. Agric. Food Chem..

[32] Sims D., Sudbery I., Ilott N.E., Heger A., Ponting C.P. (2014). Sequencing depth and coverage: Key considerations in genomic analyses. Nat. Rev. Genet..

[33] Pucker B., Kleinbölting N., Weisshaar B. (2021). Large scale genomic rearrangements in selected *Arabidopsis thaliana* T-DNA lines are caused by T-DNA insertion mutagenesis. BMC Genom..

[34] Lusk R.W. (2014). Diverse and widespread contamination evident in the unmapped depths of high throughput sequencing data. PLoS ONE.

[35] Pfeiffer F., Gröber C., Blank M., Händler K., Beyer M., Schultze J.L., Mayer G. (2018). Systematic evaluation of error rates and causes in short samples in next-generation sequencing. Sci. Rep..

[36] Xu L.N., Dong Y., Zhang J., Wang R.X., Liu H.M., Yang Q., Yang M.S. (2016). Effect of dual *Bt*-expression transformation vectors on transgene expression in tobacco. Genet. Mol. Res..

[37] Liu W., Zhao H., Miao C., Jin W. (2021). Integrated proteomics and metabolomics analysis of transgenic and gene-stacked maize line seeds. Gm Crops Food.

[38] Wang X.J., Zhang X., Yang J.T., Wang Z.X. (2018). Effect on transcriptome and metabolome of stacked transgenic maize containing insecticidal *cry* and glyphosate tolerance *epsps* genes. Plant J..

[39] Wang X., Zhang X., Yang J., Liu X., Song Y., Wang Z. (2019). Genetic variation assessment of stacked-trait transgenic maize via conventional breeding. BMC Plant Biol..

[40] Klocko A.L., Meilan R., James R.R., Viswanath V., Ma C., Payne P., Miller L., Skinner J.S., Oppert B., Cardineau G.A. (2014). Bt-Cry3Aa transgene expression reduces insect damage and improves growth in field-grown hybrid poplar. Can. J. For. Res..

[41] Podevin N., du Jardin P. (2012). Possible consequences of the overlap between the CaMV 35S promoter regions in plant transformation vectors used and the viral gene VI in transgenic plants. GM Crops Food.

[42] Han X., An Y., Zhou Y., Liu C., Yin W., Xia X. (2020). Comparative transcriptome analyses define genes and gene modules differing between two *Populus* genotypes with contrasting stem growth rates. Biotechnol. Biofuels.

[43] Quan M., Du Q., Xiao L., Lu W., Wang L., Xie J., Song Y., Xu B., Zhang D. (2019). Genetic architecture underlying the lignin biosynthesis pathway involves noncoding RNAs and transcription factors for growth and wood properties in *Populus*. Plant Biotechnol. J..

[44] Cassan-Wang H., Goué N., Saidi M.N., Legay S., Sivadon P., Goffner D., Grima-Pettenati J. (2013). Identification of novel transcription factors regulating secondary cell wall formation in *Arabidopsis*. Front. Plant Sci..

[45] Zhang J., Ge H., Zang C., Li X., Grierson D., Chen K., Yin X. (2016). EjODO1, a MYB Transcription Factor, Regulating Lignin Biosynthesis in Developing Loquat (*Eriobotrya japonica*) Fruit. Front. Plant Sci..

[46] Zhang C., Xu Y., Lu Y., Yu H., Gu M., Liu Q. (2011). The WRKY transcription factor OsWRKY78 regulates stem elongation and seed development in rice. Planta.

[47] Yu F., Huaxia Y., Lu W., Wu C., Cao X., Guo X. (2012). *GhWRKY15*, a member of the WRKY transcription factor family identified from cotton (*Gossypium hirsutum* L.), is involved in disease resistance and plant development. BMC Plant Biol..

[48] Abel S., Theologis A. (1996). Early genes and auxin action. Plant Physiol..

[49] Chen S., Zhong K., Li Y., Bai C., Xue Z., Wu Y. (2023). Evolutionary analysis of the melon (*Cucumis melo* L.) *GH3* gene family and identification of *GH3* genes related to fruit growth and development. Plants.

[50] Nakazawa M., Yabe N., Ichikawa T., Yamamoto Y.Y., Yoshizumi T., Hasunuma K., Matsui M. (2001). *DFL1*, an auxin-responsive *GH3* gene homologue, negatively regulates shoot cell elongation and lateral root formation, and positively regulates the light response of hypocotyl length. Plant J..

[51] Dewitte W., Riou-Khamlichi C., Scofield S., Healy J.M.S., Jacqmard A., Kilby N.J., Murray J.A.H. (2003). Altered cell cycle distribution, hyperplasia, and inhibited differentiation in *Arabidopsis* caused by the D-Type cyclin CYCD3. Plant Cell.

[52] Chen L., Zhao J., Song J., Jameson P.E. (2021). Cytokinin glucosyl transferases, key regulators of cytokinin homeostasis, have potential value for wheat improvement. Plant Biotechnol. J..

[53] Kajita S., Hishiyama S., Tomimura Y., Katayama Y., Omori S. (1997). Structural characterization of modified lignin in transgenic tobacco plants in which the activity of 4-Coumarate: Coenzyme a ligase 1s depressed. Plant Physiol..

[54] Liu D., Zhang J., Dong Y., Zhang X., Yang M., Gao B. (2016). Genetic transformation and expression of *Cry1Ac*-*Cry3A*-*NTHK1* genes in *Populus* × *euramericana* “*Neva*”. Acta Physiol. Plant..

[55] Li H., Durbin R. (2009). Fast and accurate short read alignment with Burrows–Wheeler transform. Bioinformatics.

[56] Love M.I., Huber W., Anders S. (2014). Moderated estimation of fold change and dispersion for RNA-seq data with DESeq2. Genome Biol..

