# Supplementary material for: Effect of T-DNA Integration on Growth of Transgenic Populus × euramericana cv. Neva Underlying Field Stands
Source: Int J Mol Sci. 2023 Aug 19;24(16):12952. doi: 10.3390/ijms241612952 (PMC10454723; doi:10.3390/ijms241612952)
Supplement: Supplementary file 1 [file ijms-24-12952-s001.zip › ijms-2536149-supplementary.pdf]

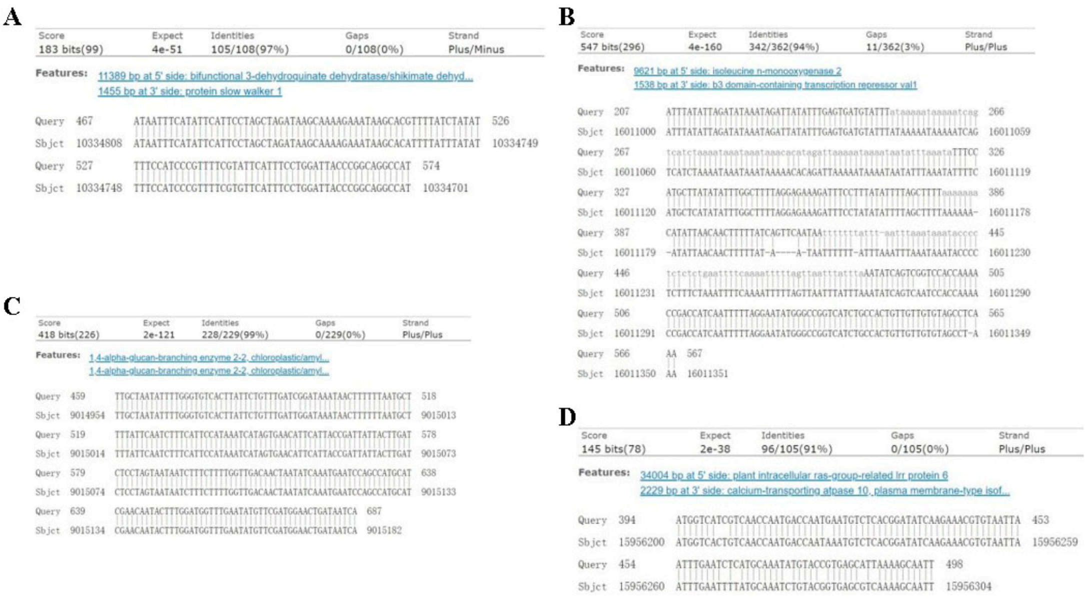


**Figure S1.** Sequence alignment of PCR products with the genome of *P. trichocarpa*. (A) Sequence alignment of PCR products of A2 line. (B) Sequence alignment of PCR products of A3 line. (C) Sequence alignment of PCR products of B3 line on Chr06. (D) Sequence alignment of PCR products of B3 line on Chr18.

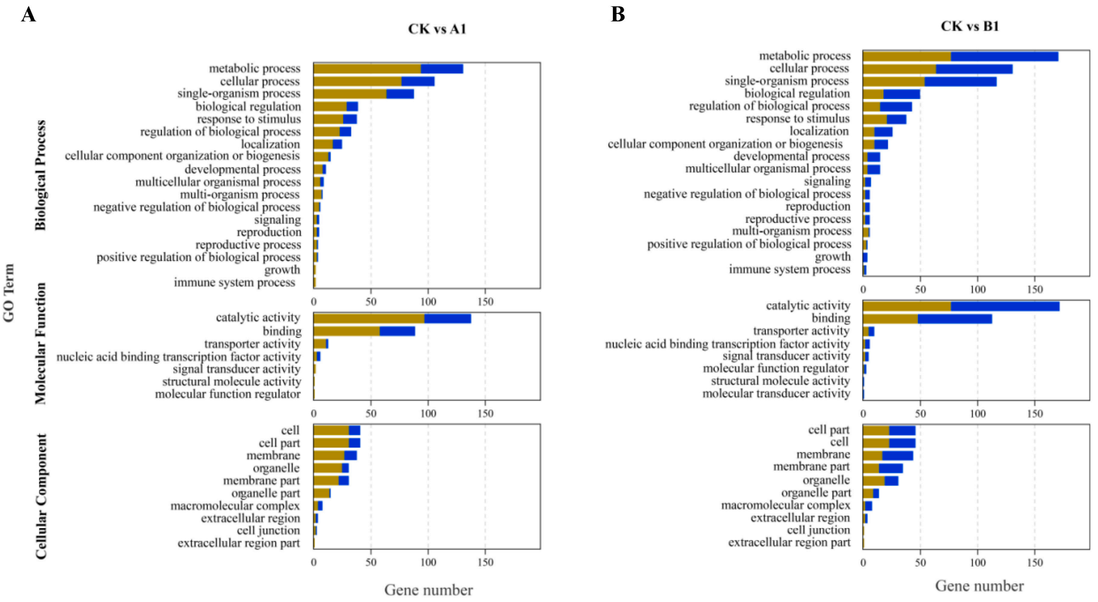


**Figure S2.** GO enrichment terms of DEGs in the A1 and B1 lines. (A) GO enrichment terms of DEGs in the A1 line. (B) GO enrichment terms of DEGs in the B1 line.
